# Inhibition Mechanism of Pancreatic K_ATP_ Channels by Centipede Toxin

**DOI:** 10.34133/research.1401

**Published:** 2026-08-17

**Authors:** Tianyi Hou, Mengmeng Wang, Yongmei Tan, Chengpeng Fan, Lei Chen

**Affiliations:** ^1^ State Key Laboratory of Membrane Biology, College of Future Technology, Institute of Molecular Medicine, Beijing Key Laboratory of Novel Antibodies and Multifunctional Conjugated Drugs, Peking University, Beijing 100871, China.; ^2^ Peking-Tsinghua Center for Life Sciences, Peking University, Beijing 100871, China.; ^3^ Academy for Advanced Interdisciplinary Studies, Peking University, Beijing 100871, China.; ^4^ School of Basic Medical Sciences, Wuhan University, Wuhan 430071, China.; ^5^ National Biomedical Imaging Center, Peking University, Beijing 100871, China.

## Abstract

The pancreatic adenosine triphosphate (ATP)-sensitive potassium (K_ATP_) channel acts as a crucial metabolic sensor, regulating insulin secretion to maintain whole-body energy homeostasis. Gain-of-function mutations in this channel lead to neonatal diabetes mellitus, a rare disorder in which certain mutants exhibit resistance to standard sulfonylurea therapy. Recent studies have identified a centipede toxin, SpTx1, as a potent inhibitor of both human pancreatic K_ATP_ channels and their gain-of-function mutants. This toxin stimulates insulin secretion, offering a promising therapeutic strategy for neonatal diabetes. However, the molecular mechanism by which SpTx1 inhibits the K_ATP_ channel has remained unclear. Here, we report the crystal structure of SpTx1 and the cryo-electron microscopy structure of the K_ATP_ channel in complex with SpTx1. The structure reveals that a single SpTx1 molecule binds to the extracellular surface of the Kir6.2 tetramer. Multiple interactions at the SpTx1–Kir6.2 interface underpin the high affinity and specificity of SpTx1 for human Kir6.2. We demonstrate that SpTx1 inhibits potassium currents by physically blocking the ion conduction pore. Furthermore, a structure-guided search identified another centipede toxin, Sm3a, as a novel K_ATP_ channel blocker. Together, these findings provide key insights into the inhibitory mechanism of centipede toxins against the human Kir6.2-containing K_ATP_ channel and establish a foundation for developing these toxins into potential therapeutics for K_ATP_ channel-related diseases.

## Introduction

The activities of adenosine triphosphate (ATP)-sensitive potassium (K_ATP_) channels are inhibited by intracellular ATP and activated by magnesium-adenosine diphosphate (Mg-ADP), thereby linking cellular metabolic status to the permeability of potassium ions on the plasma membrane [1]. As such, K_ATP_ channels adjust the cellular membrane potential according to the changes of the intracellular ATP/ADP ratio [1]. K_ATP_ channels are hetero-octamers composed of 4 central Kir6 pore subunits (either Kir6.1 or Kir6.2) and 4 regulatory sulfonylurea receptor (SUR) subunits (SUR1 or SUR2) [1]. Specifically, pancreatic K_ATP_ channels are formed by a Kir6.2 subunit and a SUR1 subunit and play a critical role in regulating insulin release [1–3]. When blood glucose levels are elevated, the cellular metabolism in pancreatic β cells is enhanced, and intracellular ATP concentration is increased, which inhibits the K_ATP_ channel [1]. This inhibition results in plasma membrane depolarization and the activation of voltage-gated calcium channels, leading to calcium influx and insulin release [1]. Therefore, K_ATP_ inhibitors such as sulfonylureas and glinides are commonly used as insulin secretagogues to block K_ATP_ channel activity and enhance insulin secretion [4].

Gain-of-function mutations in pancreatic K_ATP_ channels result in reduced ATP sensitivity and impaired insulin release in response to increased blood glucose, leading to neonatal diabetes in humans [5]. More than 80 gain-of-function mutations in the Kir6.2 channel have been found to lead to neonatal diabetes, exhibiting varying degrees of disease severity [5]. These range from transient neonatal diabetes mellitus and permanent neonatal diabetes mellitus to severe developmental delay, epilepsy, and neonatal diabetes syndrome [5]. While some patients with neonatal diabetes can achieve adequate blood glucose control with sulfonylurea therapy, others, particularly those with mutations such as L164P, C166Y, I296L, and G334D, exhibit resistance to this treatment [5].

Structural studies have shown that sulfonylureas and glinides bind to the central pocket of SUR1 in the transmembrane domain, exerting their inhibitory effects on the activity of Kir6.2 allosterically [4,6–12]. Mechanistically, they not only block the dimerization and activation of the nucleotide-binding domains of SUR1 by Mg-ADP but also recruit the N-terminal peptide of Kir6.2 (KNtp) to allosterically close the pore [4,7,10]. Mutations on SUR1 or Kir6.2 that severely disrupt these 2 mechanisms abolish the inhibitory function of sulfonylureas, rendering patients insensitive to this therapy [5]. For these sulfonylurea-resistant mutations, a promising alternative strategy to inhibit K_ATP_ channels is to directly block the Kir6.2 pore.

It has recently been discovered that centipede toxin SpTx1 can specifically inhibit human Kir6.2 (*hs*Kir6.2) with high specificity and potency [13]. Moreover, SpTx1 also blocks Kir6.2 mutants associated with neonatal diabetes that are resistant to sulfonylurea therapy [13]. Further in vivo studies have demonstrated that SpTx1 acts as a potent secondary insulin secretagogue, effectively reducing elevated blood glucose levels in diabetic mice, but it has no effect on nondiabetic mice [14], suggesting that SpTx1 as a promising reagent to treat diabetes, especially the sulfonylurea-resistant neonatal diabetes. Despite this progress, the molecular details of how SpTx1 specifically binds to and inhibits *hs*Kir6.2 remain unknown. Here, we present the crystal structure of SpTx1 and the cryo-electron microscopy (cryo-EM) structure of the SpTx1-K_ATP_ channel complex, providing insights into the inhibitory mechanism of SpTx1.

## Results 

### Crystal structure of SpTx1

**Fig. 1.**
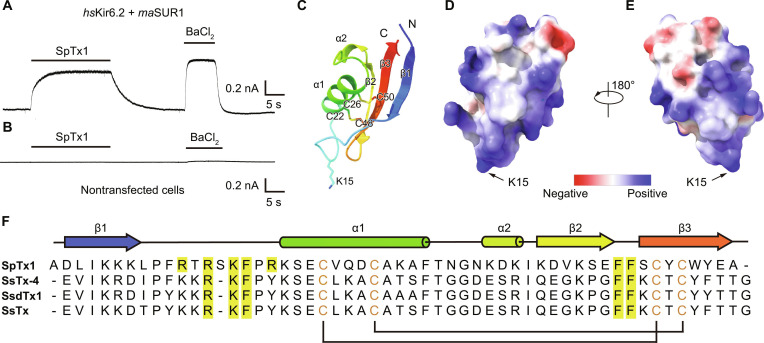
Crystal structure of SpTx1 toxin. (A) SpTx1 inhibition (1 μM) of the whole-cell current of the *hs*Kir6.2+*ma*SUR1 adenosine triphosphate (ATP)-sensitive potassium (K_ATP_) channel. Cells were clamped at −40 mV. 5 mM BaCl_2_ was used to determine the baseline. (B) Whole-cell current of the nontransfected cells performed the same as in (A). (C) Crystal structure of SpTx1, colored in rainbow. Two disulfide bonds are shown as sticks. (D) Surface electrostatic potential distribution of SpTx1, shown in red (negative) and blue (positive). SpTx1 is in the same orientation as in (C). (E) A 180°-rotated view of (D). (F) Sequence alignment of SpTx1 and 3 related toxins: SsTx-4, SsdTx1 (GenBank KC145039), and SsTx (PDB: 5X0S). α-Helices and β-sheets are represented as cylinders and arrows, respectively, and are colored according to (B). Cysteines are colored in brown, and disulfide bonds are indicated by black lines. Residues that participate in the interactions with Kir6.2 are highlighted in yellow.

We expressed the SpTx1 toxin recombinantly in *E. coli* and purified it to apparent homogeneity (Fig. S1A). Whole-cell patch clamp experiments demonstrated that purified SpTx1 can inhibit currents of the K_ATP_ channel formed by human *hs*Kir6.2 and SUR1 from *Mesocricetus auratus* (*ma*SUR1), indicating that the purified toxin is functional (Fig. 1A and B). We then crystallized SpTx1 and determined its crystal structure via x-ray crystallography at a resolution of 1.9 Å (Fig. 1C to E and Table S1). In 1 asymmetric unit, there are 6 SpTx1 monomers packed in a head-to-tail fashion, forming a hexametric ring (Fig. S1B). The formation of this hexametric assembly is likely due to the crystal packing effect, because SpTx1 migrates as a monomer on the size-exclusion chromatography (Fig. S1C). The structures of each monomer are highly similar (Fig. S1B and D). SpTx1 toxin exhibits a spearhead-shaped structure with an α-helix packing against 3 antiparallel β-sheets (Fig. 1C). Both the N-terminus and C-terminus of SpTx1 are located on the same side, at the tail of the spearhead (Fig. 1C). On the opposite side, a positively charged K15 is located at the tip of the spearhead (Fig. 1C). Two disulfide bonds are formed between C22 on α1 and C48 on β3, and between C26 on α1 and C50 on β3, further stabilizing the overall compact structure of SpTx1 (Fig. 1C). These 2 disulfide bonds were absolutely conserved in other centipede toxins that show inhibitory effect against *hs*Kir6.2 [15,16] (Fig. 1F). Notably, the surface of SpTx1 is highly hydrophilic and rich in positively charged patches (Fig. 1D and E), suggesting that polar interactions might play a crucial role in the interactions between SpTx1 and *hs*Kir6.2. Additionally, the overall structure of SpTx1 and the position of the Lys at the tip are also similar to another centipede toxin, SsTx from *Scolopendra subspinipes mutilans*, which specifically inhibits the KCNQ channel [17] (Fig. S1E and F).

### Structure determination of pancreatic K_ATP_ channel in complex with SpTx1

**Fig. 2.**
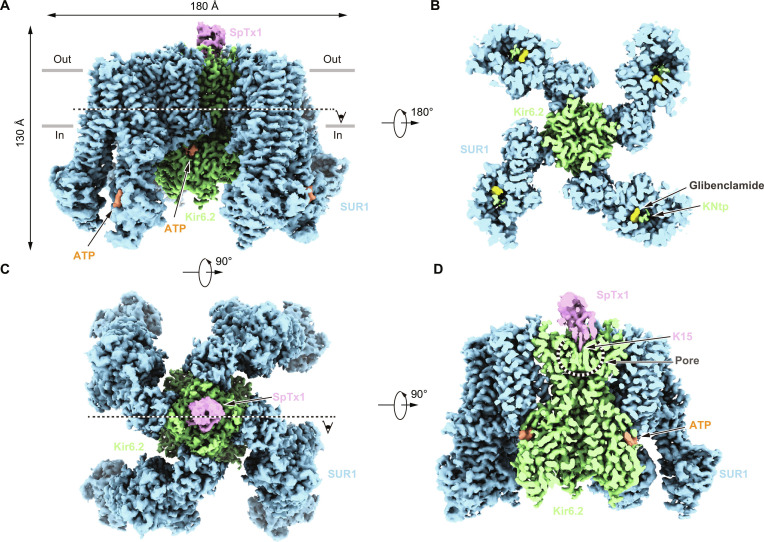
Cryo-electron microscopy (cryo-EM) structure of adenosine triphosphate (ATP)-sensitive potassium (K_ATP_) in complex with SpTx1. (A) Cryo-EM density map of K_ATP_ in complex with SpTx1, viewed from the side. The *hs*Kir6.2 subunits, *ma*SUR1 subunits, ATP, and SpTx1 are colored in green, blue, orange, and pink, respectively. (B) A bottom cut-open view, the position is indicated by the dashed line in (A), glibenclamide and N-terminal peptide of Kir6.2 (KNtp) are colored in yellow and green, respectively. (C) Cryo-EM density map of K_ATP_ in complex with SpTx1, viewed from the top. (D) A cut-open view from the side at the position of the dashed line indicated in (C), illustrating the binding of SpTx1 at the *hs*Kir6.2 pore.

To understand how SpTx1 binds the K_ATP_ channel, we sought to determine the cryo-EM structure of their complex. To express the K_ATP_ channel for structural studies with SpTx1, we fused *hs*Kir6.2 to the C-terminus of *ma*SUR1 and incorporated an PreScission Protease cleavage site upstream of *hs*Kir6.2 (Fig. S2A). This design ensured the 4:4 stoichiometry between *hs*Kir6.2 and *ma*SUR1 and facilitated the correct assembly of the channel [7,8]. Additionally, cleavage of the *ma*SUR1-*hs*Kir6.2 fusion protein by PreScission Protease released the Kir6.2 N-terminal peptide (KNtp), which is crucial for conferring sensitivity to insulin secretagogues such as glibenclamide [7,8]. We also introduced mutations Q52E in *hs*Kir6.2 and E203K in *ma*SUR1 to enhance the ATP sensitivity of the K_ATP_ channel [18] (Fig. S2A). The resulting K_ATP_ fusion construct retains the sensitivity to SpTx1 inhibition (Fig. S2B), the same as wild-type channel (Fig. 1A). Following PreScission Protease cleavage of the expressed K_ATP_ fusion protein and fractionation on size-exclusion chromatography (Fig. S2C), we supplemented the protein with purified SpTx1 toxin for cryo-EM sample preparation. We also supplemented the channel protein with ATP and glibenclamide to stabilize the channel in the ATP-bound and glibenclamide-bound closed conformation to obtain a high-resolution structure (Fig. S3). During the cryo-EM image analysis, consensus refinement using C4 symmetry yielded a map with an overall resolution of 2.74 Å (Fig. S3); however, the SUR1 ABC transporter region (SUR1_ABC_) appeared blurry, confirming its dynamic interface with the central Kir6.2 and SUR1 TMD0 region (K_ATP CORE_) [12]. Additional focused refinement on SUR_ABC_ with C1 symmetry improved the resolution to 2.87 Å (Fig. S3). In the map of SUR_ABC_, we observed the density of glibenclamide in the transmembrane domain, ATP on nucleotide-binding domain 1, and KNtp in the central cavity of *ma*SUR1 (Fig. S4). In the map of K_ATP CORE_, we identified toxin density at the extracellular mouth of the Kir6.2 channel (Fig. S3), which was absent in previous K_ATP_ maps with *hs*Kir6.2 [19,20]. However, due to the C4 averaging, the density of SpTx1 remained uninterpretable. Further symmetry expansion and focused classification with C1 symmetry on SpTx1 resulted in 2 distinct 3-dimensional classes (Fig. S3): one with no SpTx1 bound and another exhibiting prominent SpTx1 density. The class with SpTx1 bound was refined to a resolution of 2.88 Å with C1 symmetry and was subsequently merged with the SUR1_ABC_ maps to generate the composite map for model building and interpretation (Fig. 2, Figs. S3 and S4, and Table S2). In the map, we observed all of the ligands added during sample preparation, including ATP bound in the Kir6.2 subunit, ATP bound in the SUR1 subunit (Fig. 2A), and glibenclamide bound in the SUR1 subunit (Fig. 2B). We also observed discontinuous density of KNtp in the central cavity of SUR1 (Fig. S4B and C), in agreement with its dynamic nature.

### Interactions between SpTx1 and *hs*Kir6.2

**Fig. 3.**
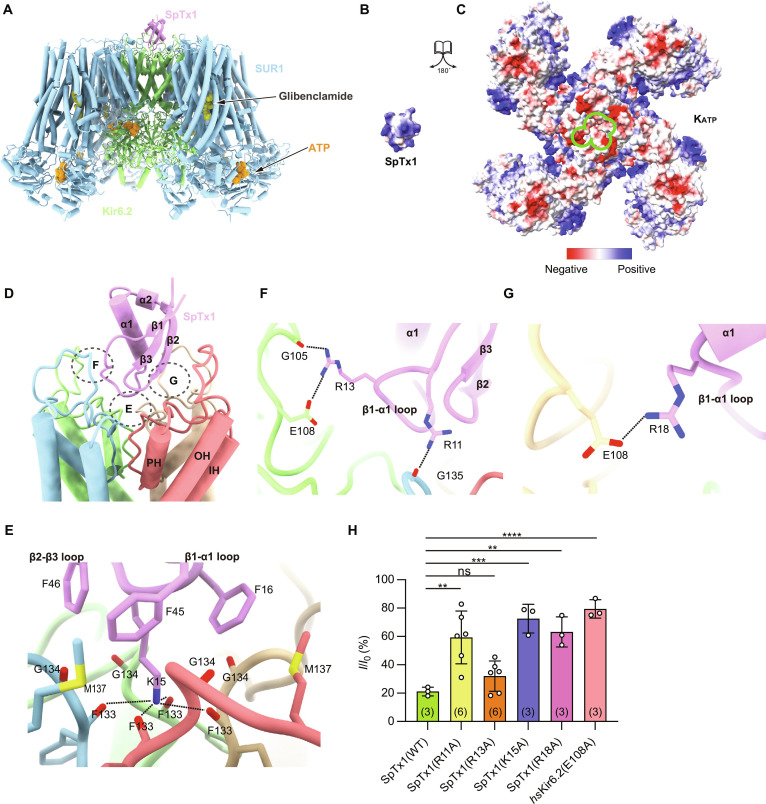
Interaction interface between SpTx1 and adenosine triphosphate (ATP)-sensitive potassium (K_ATP_). (A) Overall structure of K_ATP_ in complex with SpTx1, α-helices, and β-sheets are represented as cylinders and arrows, respectively. The ligands are shown as spheres. The *hs*Kir6.2 subunits, *ma*SUR1 subunits, ATP, glibenclamide, and SpTx1 are colored in green, blue, orange, yellow, and pink, respectively. (B and C) The open-book view of the interface between K_ATP_ (C) and SpTx1 (B) shows the electrostatic potential distribution. The binding sites of SpTx1 to K_ATP_ are circled with a green line. Negatively and positively charged patches are shown in red and blue. (D) SpTx1 and the 4 *hs*Kir6.2 subunits are shown as cartoons. Key interfaces are circled with dashed lines. SpTx1 and the *hs*Kir6.2 subunits are colored in pink, blue, green, coral, and brown, respectively. IH, inner helix; PH, pore helix; OH, outer helix. (E to G) Detailed views of the interfaces between SpTx1 and *hs*Kir6.2, as circled in (D). (H) Residual currents of *hs*Kir6.2-containing K_ATP_ channel inhibited by SpTx1 and its mutants at 1 μM concentration, determined using whole-cell patch clamp. Currents after the SpTx1 application are normalized to the currents before application. Data are presented as mean values ± SD. The numbers of independent experiments are labeled in brackets. *P* values for inhibition between SpTx1(WT) and SpTx1(R11A), SpTx1(R13A), SpTx1(K15A), and SpTx1(R18A) for the *hs*Kir6.2-containing K_ATP_ channel are 1.80 × 10^−3^, 5.99 × 10^−1^, 4.00 × 10^−4^, and 2.70× 10^−3^, respectively. The *P* value for inhibition between SpTx1(WT) for the *hs*Kir6.2-containing K_ATP_ channel and the *hs*Kir6.2(E108A)-containing K_ATP_ channel is lower than 1.0 × 10^−4^ (**P < 0.01, ***P < 0.001, ****P < 0.0001, 1-way analysis of variance [ANOVA]). ns, not significant.

The structure reveals that SpTx1 binds to the highly negatively charged surface on the extracellular side of the K_ATP_ channel (Fig. 3A to C and Fig. S4) and primarily engages with *hs*Kir6.2 subunits, in agreement with previous data showing that SpTx1 potently inhibits Kir6.2-ΔC26 [13]. SpTx1 positions its positively charged K15 at its tip into the extracellular mouth of the *hs*Kir6.2 pore (Fig. 3B to D and Movie S1). The primary amine group of K15 is situated on the 4-fold symmetry axis and near the S1 site of the pore of *hs*Kir6.2, where it interacts with the 4 backbone carbonyl groups of F133 located at the selectivity filter of *hs*Kir6.2 (Fig. 3E and Fig. S4E). Surrounding K15 are 3 phenylalanines—F16 on the β1-α1 loop and F45 and F46 on the β2-β3 loop of SpTx1—which engage with the carbonyl groups of G134 at the selectivity filter of *hs*Kir6.2 via the edges of their benzene rings and also form hydrophobic interactions with residues at the pore (Fig. 3E and Fig. S4F). Particularly, both F16 and F45 form hydrophobic interactions with M137 of 2 adjacent *hs*Kir6.2 subunits (Fig. 3E and Fig. S4G). Additionally, R11 on the β1-α1 loop of SpTx1 forms 1 hydrogen bond with the carbonyl group of G135 at the mouth of the Kir6.2 pore (Fig. 3F and S4H). Meanwhile, R18 on the β1-α1 loop of SpTx1 interacts with E108 on the S2-S3 turret of 1 *hs*Kir6.2 subunit (Fig. 3G and Fig. S4I), and R13 on the same loop further interacts with both E108 and the carbonyl group of G105 on the turret between inner helix and outer helix (OH) of the adjacent *hs*Kir6.2 subunit (Fig. 3F and Fig. S4J). To investigate the functional significance of these hydrophilic interactions, we mutated the relevant charged residues to alanines and measured the inhibitory effects of 1 μM SpTx1 mutant proteins, which is close to the saturated concentration of wild-type SpTx1 (Fig. S5). We found that the K15A mutant of SpTx1 exhibited a great loss of inhibition of the K_ATP_ channel (Fig. 3H). Similarly, the R11A, R13A, and R18A mutations in SpTx1 all reduced its inhibitory effect to some extent (Fig. 3H). Additionally, the E108A mutation in *hs*Kir6.2 conferred resistance of the K_ATP_ channel to SpTx1 inhibition (Fig. 3H). These findings confirm the crucial roles of these polar interactions and are largely consistent with previous mutagenesis results [14,15]. To understand the mechanism of high selectivity of SpTx1, we performed the sequence alignment of all Kir proteins as well as other potassium channel proteins such as KCNQ2, HCN4, and KCNH2 of *Homo sapiens*. We found that only *hs*Kir6.2 has glutamic acid at 108 position (Fig. S4N), in agreement with the high selectivity of SpTx1 toward Kir6.2.

### SpTx1 inhibits *hs*Kir6.2 by blocking its ion permeation pathway

**Fig. 4.**
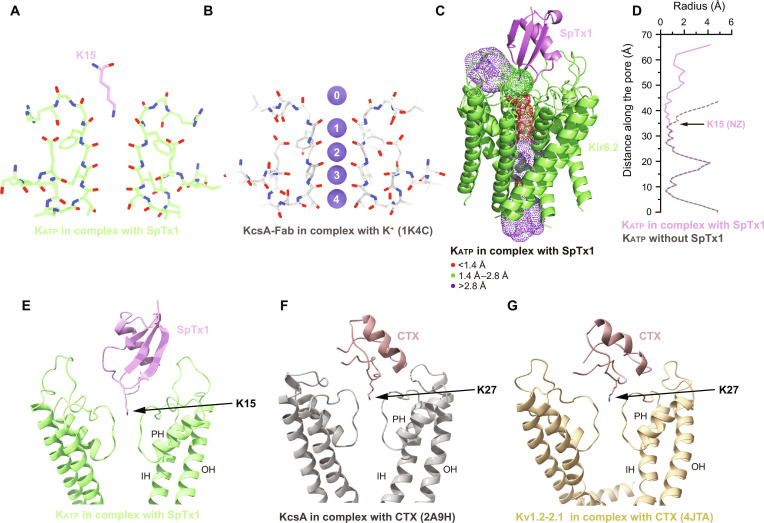
SpTx1 blocks the ion permeation pathway of *hs*Kir6.2. (A) The relative position of K15 of SpTx1 (pink) and the selectivity filter of *hs*Kir6.2 (green). Only 2 diagonal subunits of *hs*Kir6.2 are shown for clarity. (B) Potassium ion binding sites of the KcsA channel. KcsA and potassium ions are colored in gray and purple. Numbers 0 to 4 denote the ion binding sites along the selectivity filter. (C) The ion permeation pathway along the pore of *hs*Kir6.2 is shown as dots, which are colored in red, green, and purple according to the radii of <1.4, 1.4 to 2.8, and >2.8 Å. SpTx1 and *hs*Kir6.2 are shown in pink and green cartoons. (D) Pore profiles of *hs*Kir6.2 with (pink line) or without (gray dashed line) SpTx1. The position of the NZ atom of SpTx1-K15 is indicated by an arrow. (E to G) The similar block mechanism of charybdotoxin (CTX) and SpTx1 toward potassium channels. Adenosine triphosphate (ATP)-sensitive potassium (K_ATP_)_,_ KcsA, Kv1.2-2.1, SpTx1, and CTX are colored in green, gray, gold, pink, and brown, respectively. The key residues of the toxin are denoted by arrows. IH, inner helix; PH, pore helix; OH, outer helix.

K_ATP_ channel conducts potassium currents by providing an ion permeation pathway through its pore formed by 4 Kir6.2 subunits (Fig. 4A). The structure of the Kir6.2 pore is highly similar to that of other potassium channels, exemplified by the KcsA [21]. In the high-resolution crystal structure of KcsA in high potassium condition, potassium ions bind at both the extracellular mouth and 4 positions (S1 to S4) within the selectivity filter [21] (Fig. 4B). However, in the structure of K_ATP_ in complex with SpTx1, SpTx1 physically occupies not only the potassium binding site at the extracellular mouth (S0 site) but also the S1 position in the selectivity filter, precluding the binding of potassium ions at these sites (Fig. 4A). Moreover, SpTx1 also occludes the ion permeation pathway of *hs*Kir6.2 (Fig. 4A to D), preventing ion permeation (Fig. 4D). In the structure, the gate of *hs*Kir6.2 is tightly closed (Fig. 4D), in agreement with the binding of inhibitory ATP (Fig. 2D). Furthermore, we found that there is little conformational change in the extracellular regions of Kir6.2 channel upon channel opening or the binding of SpTx1 (Fig. S4K to M). Given the fact that SpTx1 can block the open *hs*Kir6.2 (Fig. 1A), we speculate that SpTx1 also interacts with *hs*Kir6.2 in the open state as observed here.

It is previously found that the potassium channel blocker charybdotoxin (CTX), derived from scorpions, also exhibits the α/β defensin fold, characterized by 3 disulfide bonds. But the structure of CTX is largely different from SpTx1, especially the position of the helix in relation to the rest of the protein (Fig. S1G). Nuclear magnetic resonance studies [22] and crystallographic studies [23] have shown that CTX also binds to the extracellular mouth of the potassium channel pore, with a lysine side chain obstructing the ion permeation pathway (Fig. 4F and G), using a mechanism similar to the SpTx1 observed here. However, the orientation of CTX and detailed interactions with potassium are largely different compared to SpTx1 with Kir6.2 (Fig. 4E to G).

### Centipede toxin Sm3a is a novel K_ATP_ channel blocker

**Fig. 5.**
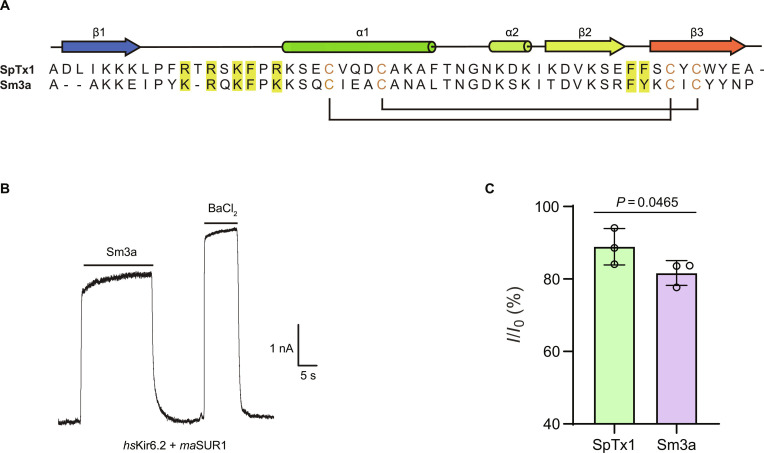
Sm3a inhibits adenosine triphosphate (ATP)-sensitive potassium (K_ATP_) channel. (A) Sequence alignment of SpTx1 and Sm3a. Secondary structures are shown the same as Fig. 1E. Residues that are important for adenosine triphosphate (ATP)-sensitive potassium (K_ATP_) inhibition are highlighted in yellow. (B) 1 μM Sm3a inhibits the whole-cell current of the *hs*Kir6.2+*ma*SUR1 K_ATP_ channel. Cells were clamped at −40 mV. BaCl_2_ (5 mM) was used to determine the baseline. (C) Residual currents of *hs*Kir6.2+*ma*SUR1 K_ATP_ channel inhibition by 200 nM SpTx1 and Sm3a, respectively, determined using whole-cell patch clamp. Currents after SpTx1 and Sm3a application are normalized to the currents before application. Data are presented as mean values ± SD. *P* value was calculated with paired *t* test.

Based on our structural and functional analysis (Fig. 3), we identified key residues within the SpTx1 toxin that are essential for inhibiting *hs*Kir6.2 (Fig. 1E). Using the SpTx1 sequence as a query, we performed a BLASTp search against the NCBI ClusteredNR database. Among the resulting hits, the toxin Sm3a from the centipede *Scolopendra morsitans* ranked top. Although Sm3a shares only 56% sequence identity with SpTx1, all key residues critical for Kir6.2 inhibition are highly conserved in Sm3a (Fig. 5A). To assess whether Sm3a also inhibits K_ATP_ channels, we recombinantly expressed and purified the toxin. Whole-cell patch-clamp recordings demonstrated that Sm3a strongly blocks currents from K_ATP_ channels composed of *hs*Kir6.2 and *ma*SUR1 subunits (Fig. 5B). Additionally, we compared the inhibitory effect of Sm3a with that of SpTx1 using 200 nM concentration, which is below the half-maximal inhibitory concentration of wild-type SpTx1. We found that the application of 200 nM Sm3a inhibited K_ATP_ channels to a similar extent as 200 nM SpTx1 (Fig. 5C), indicating comparable inhibitory potency. Given the substantial sequence similarity between SpTx1 and Sm3a, we propose that Sm3a binds to and inhibits hsKir6.2-containing K_ATP_ channels in a manner similar to SpTx1.

## Discussion 

**Fig. 6.**
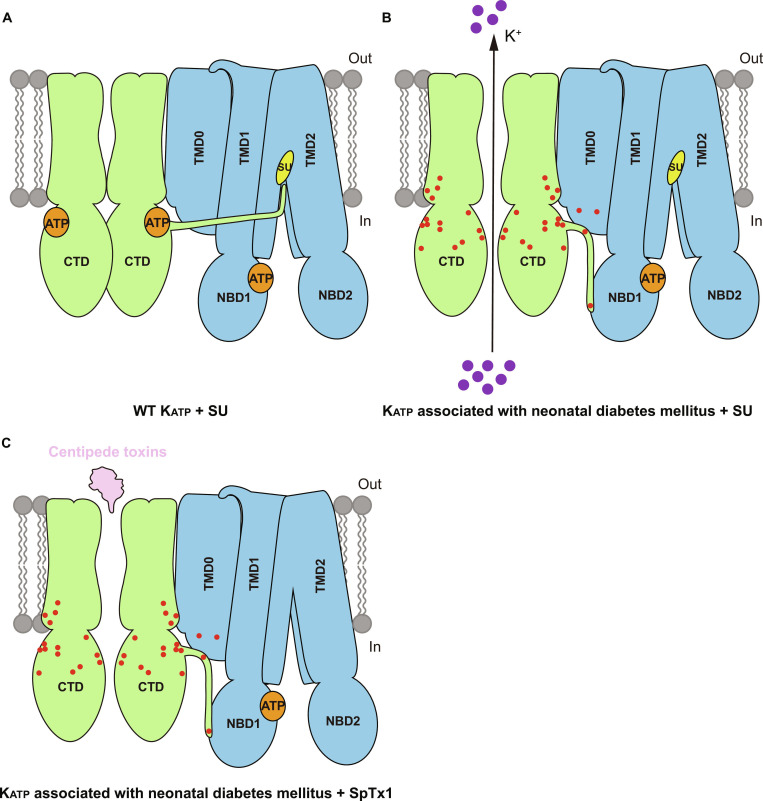
Inhibition mechanism of SpTx1 to adenosine triphosphate (ATP)-sensitive potassium (K_ATP_) channel. (A) Wild-type K_ATP_ channels can be effectively inhibited by ATP and sulfonylurea (SU). ATP, SU, Kir6.2, and SUR1 are colored in orange, yellow, green, and blue, respectively. Only 2 subunits of Kir6.2 and 1 subunit of SUR1 are shown for clarity. SU recruits the N-terminal peptide of Kir6.2 (KNtp) to allosterically close the pore of Kir6.2. SU also inhibits the closure of SUR1 subunits. (B) Some neonatal diabetes mutants of the K_ATP_ channel are constitutively active and resistant to ATP and sulfonylurea inhibition. The positions of mutations are indicated with red dots. (C) SpTx1 can effectively block the permeation pathway of K_ATP_ channels, including those sulfonylurea-resistant neonatal diabetes mutants. NBD1, nucleotide-binding domain 1; CTD, cytosolic domain; TMD0, transmembrane domain 0.

The structure of SpTx1 determined here reveals a characteristic 2-disulfide bond-stabilized α/β defensin fold, which is commonly found in centipede toxins [24]. This structural motif is notable for its stability and functionality, allowing these peptides to interact effectively with various biological targets [17]. The elucidation of complex structure between SpTx1 and *hs*Kir6.2-containing K_ATP_ channels provides critical insights into the molecular mechanisms underpinning the inhibitory action of SpTx1. It is known that several gain-of-function mutations associated with neonatal diabetes, located in either Kir6.2 or SUR1, confer resistance to inhibition by ATP or sulfonylureas (Fig. 6A and B) [5]. The binding site of SpTx1, however, is on the extracellular side of *hs*Kir6.2, distal from the common neonatal diabetes-associated mutations (Fig. 6C). This distinct binding mode explains SpTx1’s broad inhibitory effect across diverse clinical K_ATP_ channel mutants.

Moreover, the molecular targets of defensin fold centipede toxins are diverse, many of which target different potassium channels [25]. The structure mechanism of interactions between Kir6.2 and SpTx1 could serve as a paradigm for investigating other defensin fold toxin–channel interactions, potentially leading to the discovery of novel inhibitors or modulators for clinical applications. Indeed, structure-based sequence search aids the identification of Sm3a as a new inhibitor of K_ATP_ channel (Fig. 5).

Importantly, SpTx1 and K_ATP_ interact at the extracellular side of the Kir6.2 subunit, and neither the N- nor the C-termini of SpTx1 is involved in its binding to Kir6.2. As a result, there is a unique opportunity to explore protein fusions to either the N- or C-terminus of SpTx1 without compromising its functionality. This opens avenues for new modifications of SpTx1, such as fusing SpTx1 with proteins that target endocytosis receptors to facilitate the selective degradation of gain-of-function K_ATP_ mutants [26,27]. Targeting these mutants through the lysosomal degradation pathway might represent a novel therapeutic strategy, leveraging the specificity of SpTx1’s binding properties.

In conclusion, the structural and functional insights gained from the study of SpTx1-K_ATP_ complex not only enhance our understanding of the mechanism of centipede toxins but also lay the groundwork for potential therapeutic modifications of these centipede toxins to enhance their potency or broaden its application scope.

## Materials and Methods 

### Constructs

 The coding sequences of the matured form of SpTx1 and Sm3a (P0DQC2.1) were synthesized and inserted into a pET-based N-terminally His-tagged vector (pPH) with tobacco etch virus protease cleavage site to generate the toxin expression vectors.

The coding sequences of maSUR1 (E203K) and hsKir6.2 (Q52E) are linked by a long linker including a PreScission protease cleavage site (​VDG​SGS​GSG​SAA​GSG​SGS​GSG​SGA​AGS​GSG​SGS​GSG​AAALELEVLFQGP). The initial methionine of *hs*Kir6.2 after the linker was removed. The open reading frame of fusion protein was inserted into a modified BacMam vector with C-terminal green fluorescent protein and 2 Strep tags [28].

### Expression and purification of toxin

The expression vector for the toxins were transformed into Rosetta-gami 2(DE3) competent cells. Protein expression was induced by adding 1 mM isopropyl β-D-1-thiogalactopyranoside for 3 h at 37 °C in Luria–Bertani medium. Collected bacterial cells were resuspended in 20 mM tris (pH 7.5), 150 mM NaCl, 1 mM phenylmethylsulfonyl fluoride and were disrupted via sonication. The lysate was centrifuged at 30,000g for 1.5 h, after which the supernatant was applied to a Talon affinity column and eluted using 250 mM imidazole. The eluted protein was subjected to tobacco etch virus protease digestion to remove the purification tag. After cleavage, contaminant proteins were precipitated using methanol, yielding highly purified toxin protein, which was subsequently purified via SP ion-exchange chromatography to remove residual methanol. Final purification was performed using a Superdex 75 size-exclusion column running in 10 mM tris (pH 7.5) and 150 mM NaCl. Protein was concentrated to 20 mg/ml for crystallization.

### Crystallization, x-ray data collection, and structural determination

Protein was mixed with 0.1 M succinic acid (pH 5.5), 0.05 M ammonium sulfate, and 30% (v/v) pentaerythritol ethoxylate (15/4 EO/OH) with a 1/1 ratio at room temperature in a hanging drop crystallization setup. Crystal was harvested using 0.1 M succinic acid (pH 5.5), 0.05 M ammonium sulfate, 30% (v/v) pentaerythritol ethoxylate (15/4 EO/OH), and 10% ethylene glycol as cryoprotectant and flash frozen in liquid nitrogen.

Native datasets of SpTx1 were collected at Shanghai Synchrotron Radiation Facility beamline BL17U at 0.97918 Å. Diffraction images were processed using HKL3000 [29] and converted to a mtz file using CCP4 [30]. SpTx1 structure predicted by AlphaFold2 [31] was used as the search model for molecular replacement using Phaser [32]. The model was rebuilt manually using COOT [33] and was refined using Phenix [34]. The final model contains 99.68% favored and 0.32% allowed residues but no outliers on the Ramachandran plot.

### Expression and purification of K_ATP_ channel

K_ATP_ channels were expressed as described previously, and the purification process was carried out with minor modification [7,35]. For protein purification, membrane pellets were homogenized in tris buffer saline (TBS, 20 mM tris-HCl [pH 7.5], and 150 mM NaCl) and solubilized in TBS with 1% digitonin (Calbiochem), supplemented with protease inhibitors (1 mg/ml leupeptin, 1 mg/ml pepstatin, 1 mg/ml aprotinin, and 1 mM phenylmethylsulfonyl fluoride), 5 μM glibenclamide, 1 mM ATP, 1 mM EDTA, 2.5 mM MgCl₂, and 2.5 mM CaCl₂, for 30 min at 4 °C. Insoluble materials were removed after centrifugation at 100,000g for 30 min, and the supernatant was loaded onto a 5-ml column packed with Streptactin 4FF resin (Smart Lifesciences). Strep column was first washed by buffer A (5 μM glibenclimide, 1 mM ATP, 1 mM EDTA, and 0.1% digitonin in TBS), then 1 mM ATP and 2.5 mM MgCl₂ in buffer A, and finally in buffer A again. K_ATP_ protein was eluted with 10 mM desthiobiotin in buffer A. Protein was then digested with PreScission protease overnight, concentrated, and purified using a Superose 6 column running in buffer A. Peak fractions were collected and concentrated to *A*_280_ (absorbance at 280 nm) = 15 (estimated as 15 μM K_ATP_ octamers) for cryo-EM sample preparation.

### Cryo-EM sample preparation

K_ATP_ octamers were supplemented with 200 μM glibenclimide and 1 mM ATP, along with 100 μM SpTx1. The final K_ATP_ protein concentration was estimated to be 9 μM octamer in solution. Cryo-EM grids were prepared with GIG R1/1 holey carbon grids, which were glow-discharged for 120 s. A 2.5-μl K_ATP_ octamer sample was applied to the glow-discharged grid, and then the grid was blotted at blotting force in level 2 for 2 s at 100% humidity and 20 °C, before plunge-frozen into the liquid ethane.

### Cryo-EM data collection

Cryo-EM data collection was performed on a Titan Krios microscope (Thermo Fisher Scientific) operating at 300 kV. Images were acquired using a Gatan K2 direct electron detector mounted post a quantum energy filter with a 20-eV slit width. Super-resolution mode was employed with a calibrated pixel size of 1.324 Å at the specimen level, and data acquisition was controlled via SerialEM software. Defocus values were systematically varied between −1.3 and −1.8 μm to optimize contrast transfer. A dose rate of 8 e^−^/s/Å^2^ was applied, with a cumulative electron exposure of 50 e^−^/Å^2^. Each 12-s exposure was dose-fractionated into 50 frames to facilitate motion correction during image processing.

### Cryo-EM image processing and model building

A total of 5,655 movies were corrected for beam-induced motion, dose-weighted, and 2-fold binned to a pixel size of 1.324 Å using MotionCor2 [36]. The contrast transfer function parameters of micrographs were estimated using patch contrast transfer function in cryoSPARC [37]. Iterative 2-dimensional and 3-dimensional classification generated 548,390 particles with 256 box size which were refined to 2.74-Å overall resolution using nonuniform refinement function in cryoSPARC [37]. Symmetry expansion, particle subtraction, and local refinement of the SUR1_ABC_ regions yielded a local map of 2.87-Å resolution. Symmetry expansion and iterative focused classification of SpTx1 region using C1 symmetry resolved 2 classes: One has SpTx1 bound and the other has no SpTx1 bound. The class with SpTx1 bound was locally refined using C1 symmetry focusing on the K_ATP CORE_ region to obtain a map at 2.88-Å resolution. This map of K_ATP CORE_ was combined with the map of SUR1_ABC_ to obtain a composite map for model building and interpretation. The structures of SpTx1 and K_ATP_ channel in complex with repaglinide (Protein Data Bank [PDB] ID: 6JB1) were docked into the composite map. The model was rebuilt manually using COOT [33] and was refined using Phenix [34].

### Electrophysiology

K_ATP_ constructs were transfected into FreeStyle 293-F cells using polyethylenimine at a cell density of 1 × 10^6^ cells/ml. Cells were cultured in FreeStyle 293 Expression Medium with 1% fetal bovine serum for 24 h before recording. Currents were recorded using whole-cell mode at −40 mV in the pipette and an Axon-patch 200B amplifier (Axon Instruments, USA). Patch electrodes were pulled by a horizontal microelectrode puller (P-1000, Sutter Instrument Co, USA) to tip resistance of 2.0 to 3.0 MΩ. Pipette solution containing 107 mM KCl, 1.2 mM MgCl_2_, 1 mM CaCl_2_, 10 mM EGTA, 5 mM Hepes (pH 7.2, KOH), and bath solution containing 40 mM KCl, 100 mM NaCl, 2.6 mM CaCl_2_, 1.2 mM MgCl_2_, and 5 mM Hepes (pH 7.4, NaOH) were used for measuring inhibitory effect of wild-type and mutant toxins. Application of 5 mM BaCl_2_ in bath solution was used to block the K_ATP_ currents to determine the baseline. Data analysis was carried out using GraphPad Prism 8.0 or 10.4.0.

### Quantification and statistical analysis

Global resolution estimations of cryo-EM density maps are based on the 0.143 Fourier Shell Correlation criterion [38]. The local resolution was estimated using cryoSPARC. The number of independent experiments (*N*) and the relevant statistical parameters for each experiment (such as mean or SD) are described in the figure legends. No statistical methods were used to predetermine sample sizes.

## Data Availability

Crystal structure of SpTx1 has been deposited into PDB under the ID code: 9KGA. Cryo-EM maps and the atomic coordinate of the SpTx1-K_ATP_ complex have been deposited in the EMDB and PDB under the ID code EMDB: EMD-62322 and PDB ID: 9KGL.
