# Supplementary material for: Inhibition Mechanism of Pancreatic KATP Channels by Centipede Toxin
Source: Research (Wash D C). 2026 Aug 17;9:1401. doi: 10.34133/research.1401 (PMC13478508; doi:10.34133/research.1401)
Supplement: Supplementary file 1 [file research.1401.f1.zip › Supplementary_Figs.pdf]

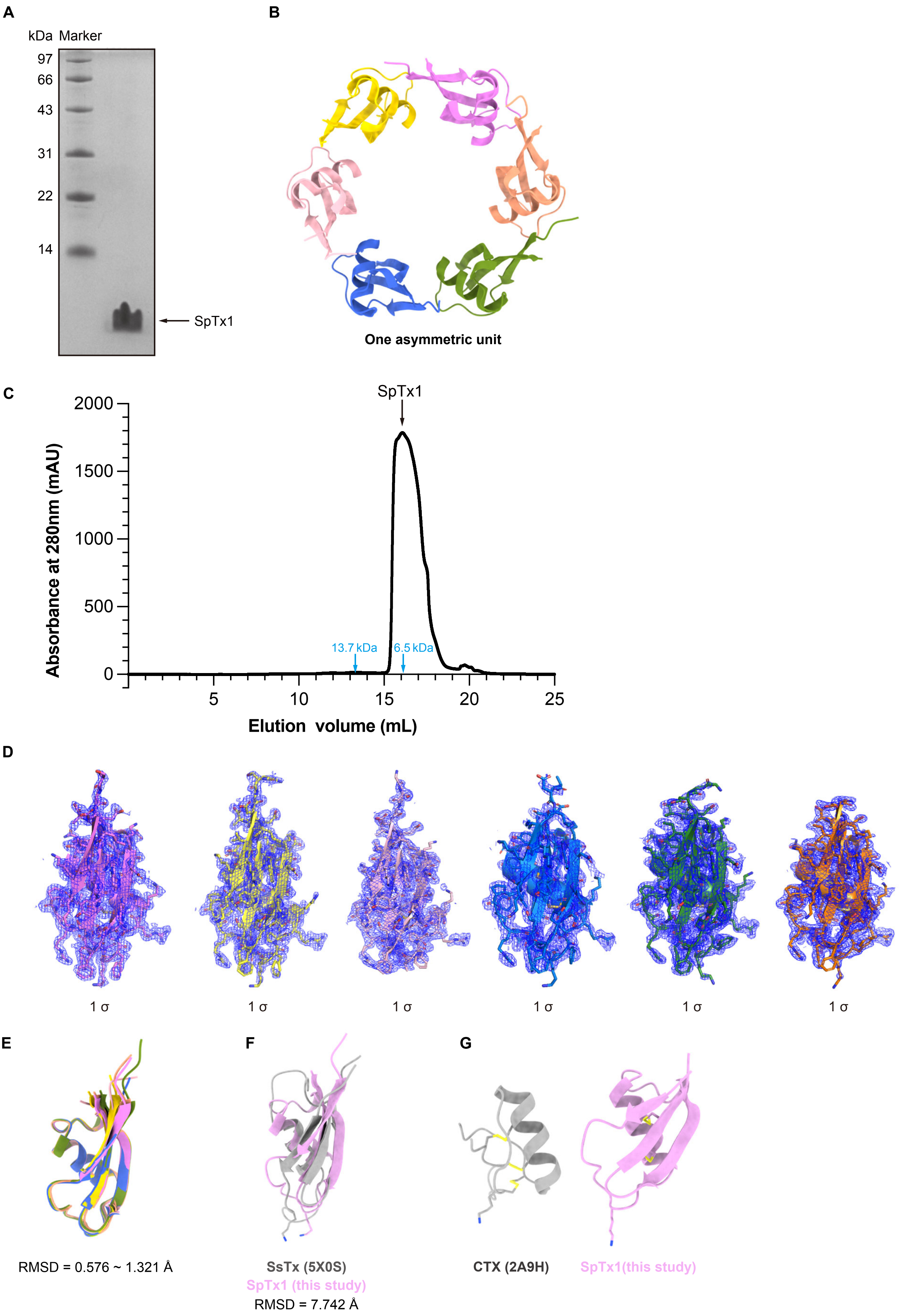

Figure S1. Crystal structure of SpTx1.

(A) Coomassie blue-stained SDS-PAGE of SpTx1 used for crystallization.

(B) One asymmetric unit of SpTx1 is shown as a hexametric ring.

(C) A representative trace of size exclusion chromatography (SEC) of the SpTx1 protein on a Superdex 75 Increase 10/300 GL column. The peak positions of molecular weight markers provided in the column instruction manual are denoted by blue arrows.

(D) Density of each monomer SpTx1 in (B).

(E) Aligned monomers with root mean square deviation 0.576-1.321Å.

(F) Structural comparison between SpTx1 and SsTx with root mean square deviation 7.742Å, SsTx and SpTx are colored in gray and pink, respectively.

(G) CTX and SpTx1 are colored in gray and pink, respectively. Disulfide bonds are shown as sticks.

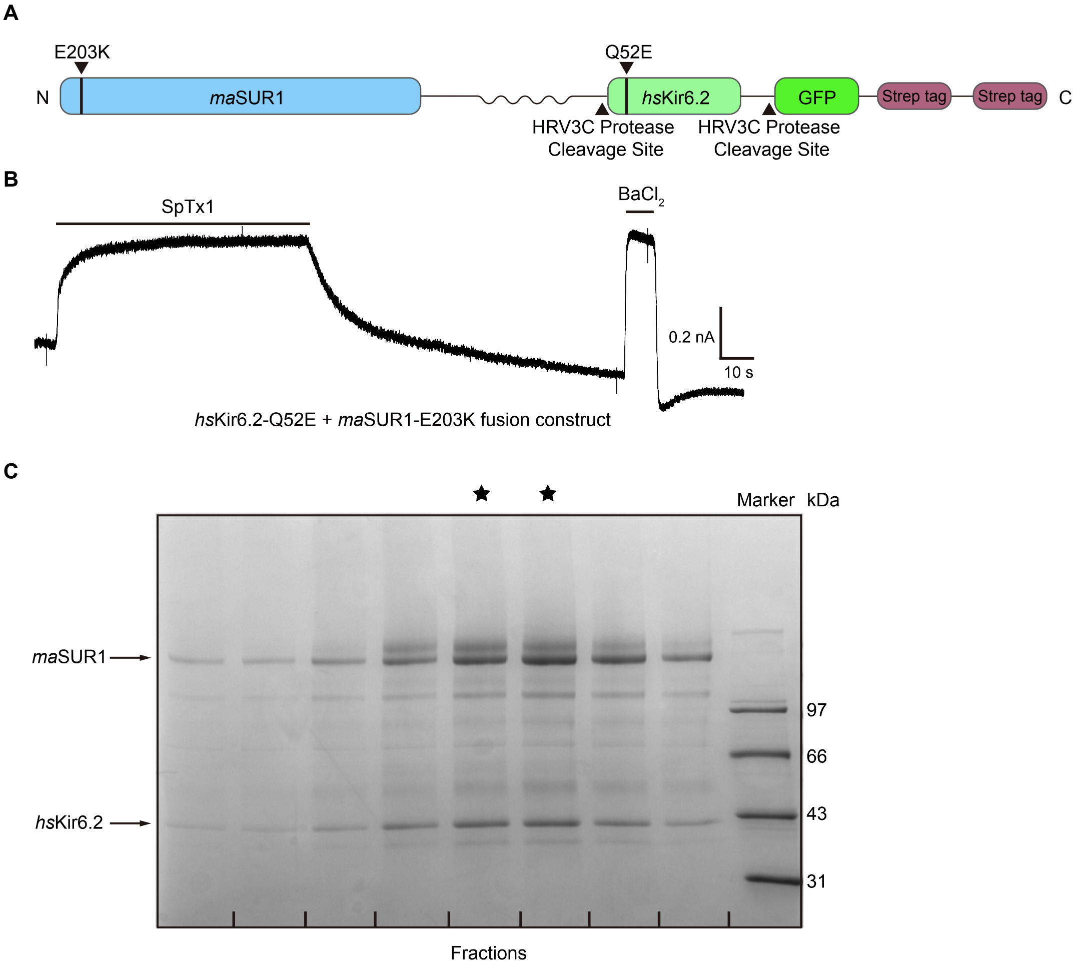

Figure S2. Protein expression and purification of KATP.

(A) The design of the KATP fusion construct used for Cryo-EM. *ma*SUR1, *hs*Kir6.2, GFP, and Strep-tag are colored in blue, pale green, green, and brown, respectively. A black wavy line represents the linker between *ma*SUR1 and *hs*Kir6.2. PreScission Protease cleavage sites and mutation sites are indicated by black triangles.

(B) Inhibition of the KATP fusion channel by 1 μM SpTx1, determined using whole-cell patch clamp.

(C) Coomassie blue-stained SDS-PAGE of the KATP protein sample from size-exclusion chromatography. Fractions labeled with asterisks were pooled for cryo-EM sample preparation.

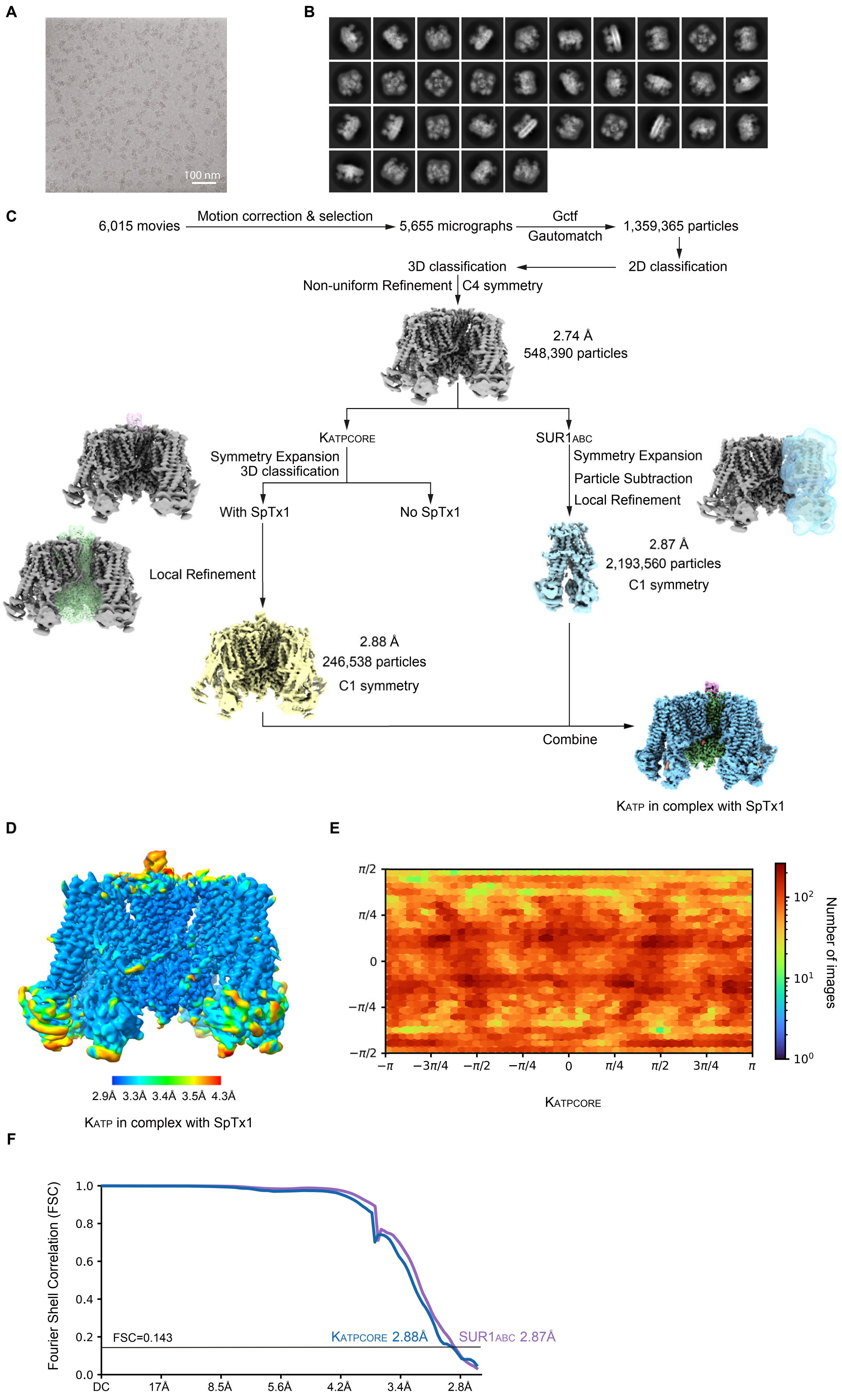

Figure S3. Workflow for cryo-EM data processing of KATP in complex with SpTx1.
(A) Representative image from a dataset consisting of 6,015 motion-corrected micrographs.
(B) Two-dimensional class averages.
(C) Cryo-EM image processing workflow.
(D) Local resolution map of the composite map.
(E) Angular distribution of the KATPCORE.
(F) Resolution estimation of the KATPCORE and SUR1ABC map, based on the gold-standard FSC 0.143 cut-off criterion.

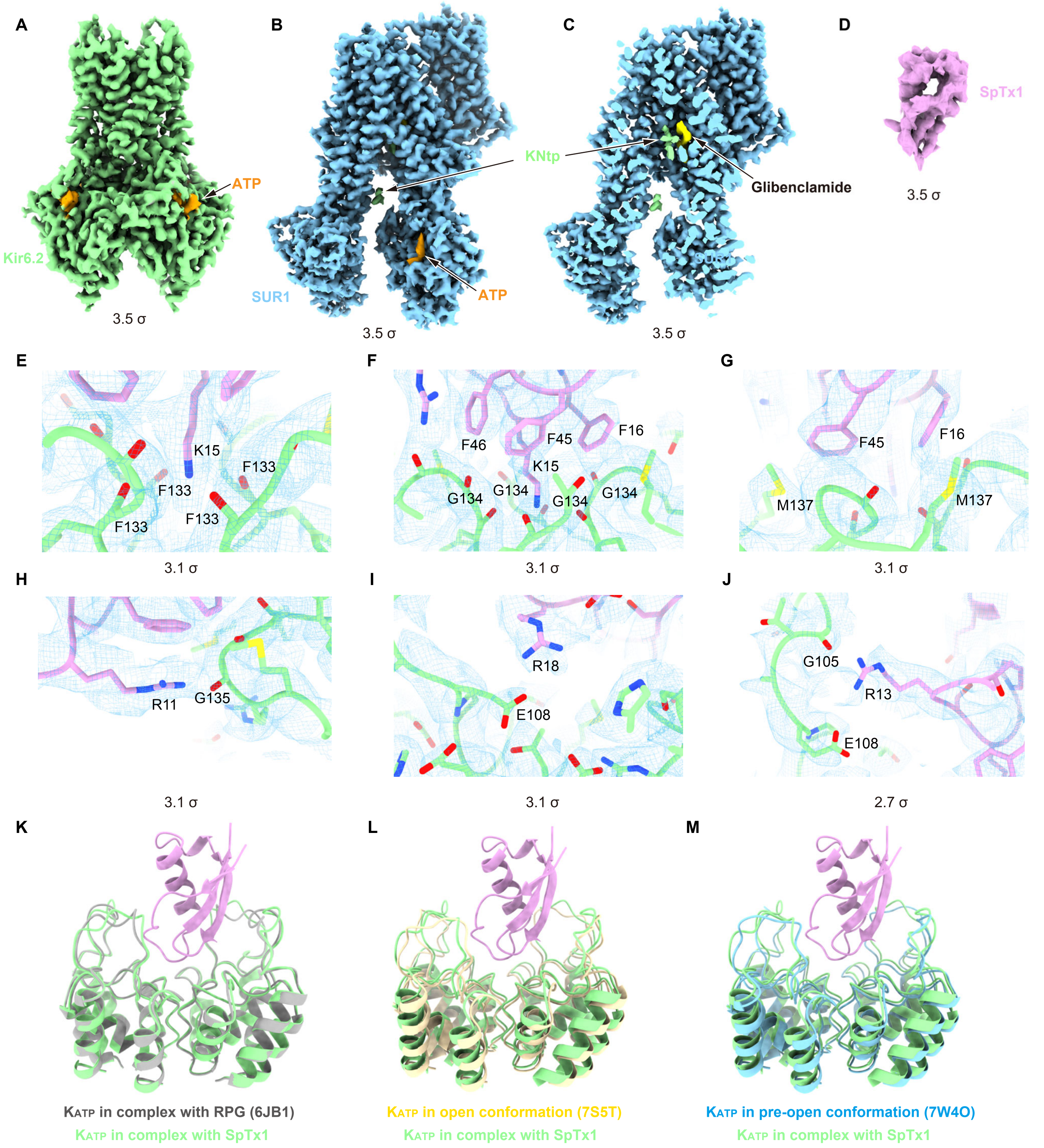

N

PH IH

```
hsKir6.2  103 S-----------------EGTAEPC----VTSIHSFSSAFLFSIEVQVTIGFGGRMVTEECPLAILILIVQN 153
hsKir1.1  111 -EFHP-------------SANHTPC----VENINGLTSAFLFSLETQVTIGYGFRCVTEQCATAIFLLIFQS 164
hsKir2.1  116 SK------------------EGKAC----VSEVNSFTAAFLFSIETQTTIGYGFRCVTDECPIAVFMVVFQS 165
hsKir2.2  115 AEGR----------------GRTPC----VMQVHGFMAAFLFSIETQTTIGYGLRCVTEECPVAVFMVVAQS 166
hsKir2.3   90 SPGVPAAGGPAAGGGGAAPVAPKPC----IMHVNGFLGAFLFSVETQTTIGYGFRCVTEECPLAVIAVVVQS 157
hsKir2.4  121 PP------------------PPAPC----FSHVASFLAAFLFALETQTSIGYGVRSVTEECPAAVAAVVLQC 170
hsKir3.1  115 AHVG----------------NYTPC----VANVYNFPSAFLFFIETEATIGYGYRYITDKCPEGIILFLFQS 166
hsKir3.2  124 IEDP----------------SWTPC----VTNLNGFVSAFLFSIETETTIGYGYRVITDKCPEGIILLLIQS 175
hsKir3.3   92 LEDT----------------AWTPC----VNNLNGFVAAFLFSIETETTIGYGHRVITDQCPEGIVLLLLQA 143
hsKir3.4  121 VGDQ----------------EWIPC----VENLSGFVSAFLFSIETETTIGYGFRVITEKCPEGIILLLVQA 172
hsKir4.1   98 -ELDP-------------PANHTPC----VVQVHTLTGAFLFSLESQTTIGYGFRYISEECPLAIVLLIAQL 151
hsKir5.1  105 DP------------------DITPC----VDNVHSFTGAFLFSLETQTTIGYGYRCVTEECSVAVLMVILQS 154
hsKir6.1  104 YMEKS--------GMEKSGLESTVC----VTNVRSFTSAFLFSIEVQVTIGFGGRMMTEECPLAITVLILQN 163
hsKir7.1   88 DHDAP-------------PENHTIC----VKYITSFTAAFSFSLETQLTIGYGTMFPSGDCPSAIALLAIQM 142
hsKCNQ2   250 ---------VY----------LAE--KGENDHFDTYADALWWGLITLTTIGYGDKYPQTWN-GRLLAATF-T 298
hsHCN4    449 ---C----WVS----------INNMVNN--SWGKQYSYALFKAMSHMLCIGYGRQAPVGMS-DVWLTMLS-M 499
hsKCNH2   581 SRIG----WLH-NLGDQIGKPYNSSGLGGPSIKDKYVTALYFTFSSLTSVGFGNVSPNTNS-EKIFSICV-M 645
```

Figure S4. Local cryo-EM densities of KATP in complex with SpTx1.

(A) Density map of the *hs*Kir6.2 subunit, contoured at 3.5 σ, viewed from the side.
(B) Density map of the *ma*SUR1 subunit, contoured at 3.5 σ, viewed from the side.
(C) Cut-open view of (B).
(D) Density map of SpTx1, contoured at 3.5 σ, viewed from the side.
(E-J) Close-up views of the key interfaces between SpTx1 and *hs*Kir6.2. SpTx1 is colored in pink, hsKir6.2 is colored in green, and the density map is shown as a blue mesh, contoured at 3.1 σ and 2.7 σ.
(K-M) Structure of KATP in complex with SpTx1 compared with the SpTx1-free closed state (K), the open state (L), and the pre-open state (M) KATP, colored in green, gray, yellow, and blue, respectively.
(N) Sequence alignment of all members of human Kirs, KCNQ2, HCN4 and KCNH2. α-helices are represented as green cylinders. Key residues are indicated by a yellow background.

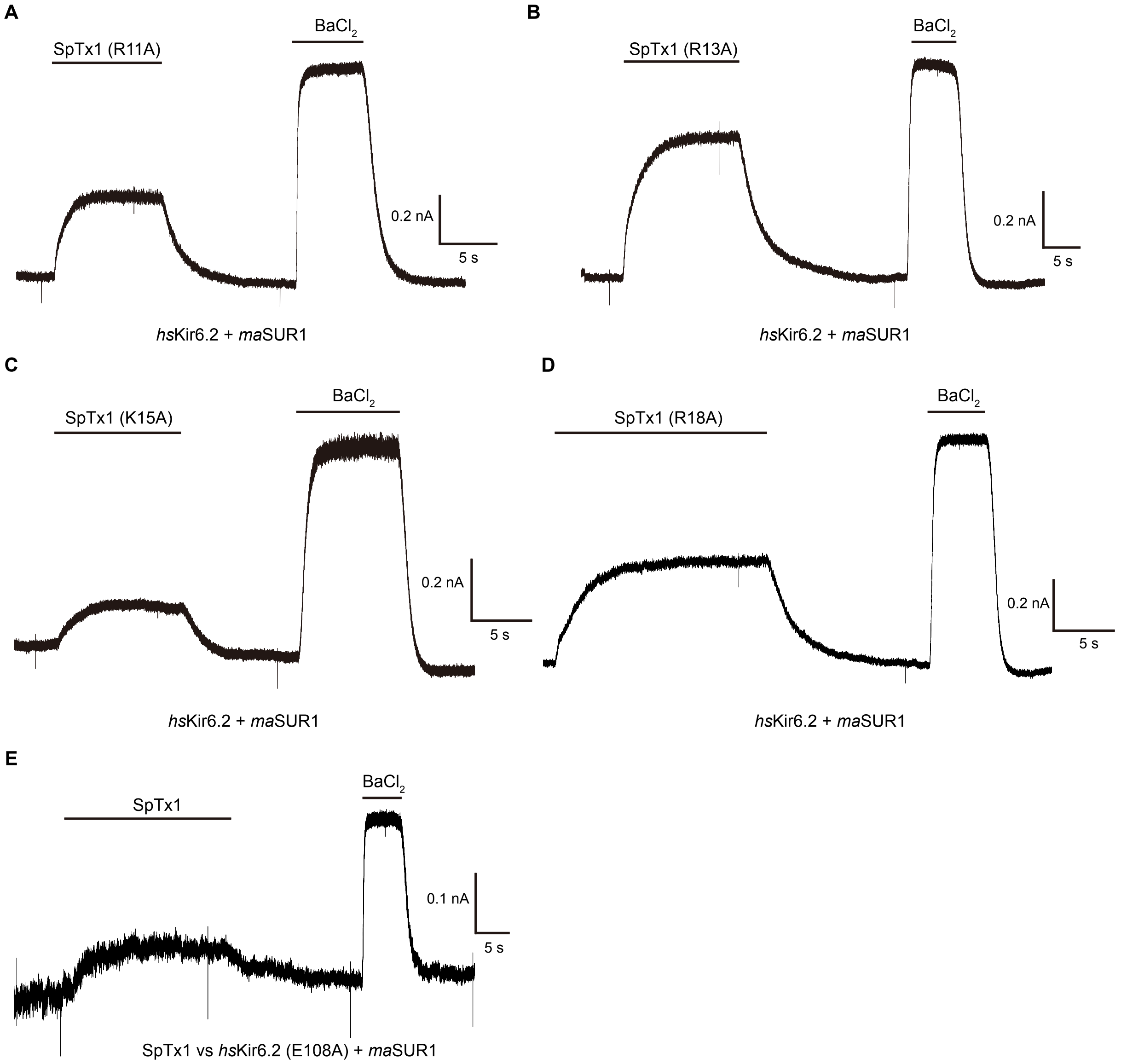

Figure S5. Representative whole-cell currents of KATP inhibition by 1 μM various SpTx1 mutants.
(A) Inhibition of the wild-type *hs*Kir6.2+*ma*SUR1 KATP channel by the SpTx1 R11A mutant.
(B) Inhibition of the wild-type *hs*Kir6.2+*ma*SUR1 KATP channel by the SpTx1 R13A mutant.
(C) Inhibition of the wild-type *hs*Kir6.2+*ma*SUR1 KATP channel by the SpTx1 K15A mutant.
(D) Inhibition of the wild-type *hs*Kir6.2+*ma*SUR1 KATP channel by the SpTx1 R18A mutant.
(E) Inhibition of the *hs*Kir6.2 E108A mutant + *ma*SUR1 KATP channel by wild-type SpTx1.

Table S1 Data collection and refinement statistics of SpTx1 (molecular replacement)

	SpTx1 (PDB ID: 9KGA)
**Data collection**	
Space group	*P3₁21*
Cell dimensions	
a, b, c (Å)	60.49, 60.49, 169.85
α, β, γ (°)	90.00, 90.00, 120.00
Resolution (Å)	50-1.90 (1.93-1.90)*
R_{sym} or R_{merge}	0.11 (0.75)
I / σI	22.50 (3.33)
Completeness (%)	100.00(100.00)
Redundancy	9.2 (8.8)
**Refinement**	
Resolution (Å)	44.58-1.9
No. reflections	29,121
R_{work} / R_{free}	0.2031/0.2138
No. atoms	3,000
Protein	2,702
Ligand/ion	0
Water	298
B-factors (Å^2)	28.54
Protein	27.99
Ligand/ion	
Water	33.51
R.m.s. deviations	
Bond lengths (Å)	0.01
Bond angles (°)	0.81

*Values in parentheses are for highest-resolution shell.

Table S2 Cryo-EM data collection, refinement and validation statistics

	Composite map (EMD-62322) (PDB 9KGL)	Focused map of $KATP_{core}$ (EMD-62311)	Focused map of $SUR1_{ABC}$ (EMD-62317)
**Data collection and processing**			
Magnification		38,000 ×	
Voltage (kV)		300	
Electron exposure (e–/Å^2)		50	
Defocus range (set, µm)		-1.5 to -1.8	
Pixel size (Å)		1.324	
Symmetry imposed		C1	
Initial particle images (no.)		1,595,249	
Final particle images (no.)	N/A	246,583	2,193,560*
Map resolution (Å)	N/A	2.88	2.87
FSC threshold	N/A	0.143	0.143
Map resolution range (Å)	N/A	338.94-2.88	338.94-2.87
**Refinement**			
Initial model used (PDB code)	6JB1		
Model resolution (Å)	3.1		
FSC threshold	0.5		
Model resolution range (Å)	338.94-3.1		
Map sharpening *B* factor (Å^2)		112.4	147.9
Model composition			
Non-hydrogen atoms	55,831		
Protein residues	7,074		
Ligands	12		
B factors (Å^2)			
Protein	69.12		
Ligand	58.30		
R.m.s. deviations			
Bond lengths (Å)	0.005		
Bond angles (°)	0.996		
Validation			
MolProbity score	1.65		
Clashscore	5.66		
Poor rotamers (%)			
Ramachandran plot			
Favored (%)	97.61		
Allowed (%)	2.27		
Disallowed (%)	0.11		

* The particles are expended using C4 symmetry for the local refinement of SUR1.
